# The relation of representational competence and conceptual knowledge in female and male undergraduates

**DOI:** 10.1186/s40594-023-00435-6

**Published:** 2023-06-21

**Authors:** Peter A. Edelsbrunner, Sarah Malone, Sarah I. Hofer, Stefan Küchemann, Jochen Kuhn, Roman Schmid, Kristin Altmeyer, Roland Brünken, Andreas Lichtenberger

**Affiliations:** 1 Department of Humanities, Social and Political Sciences, ETH Zurich, RZ H16, Claussiusstrasse 59, 8092 Zurich, Switzerland; 2 Faculty of Psychology and Education, LMU Munich, Munich, Germany; 3 Department of Education, Saarland University, Saarbrücken, Germany; 4 Faculty of Physics, LMU Munich, Munich, Germany; 5 Department of Physics, ETH Zurich, Zurich, Switzerland

**Keywords:** Conceptual understanding, Representational competence, Multiple external representations, Latent variable modeling, Gender

## Abstract

**Background:**

Representational competence is commonly considered a prerequisite for the acquisition of conceptual knowledge, yet little exploration has been undertaken into the relation between these two constructs. Using an assessment instrument of representational competence with vector fields that functions without confounding topical context, we examined its relation with *N* = 515 undergraduates’ conceptual knowledge about electromagnetism.

**Results:**

Applying latent variable modeling, we found that students’ representational competence and conceptual knowledge are related yet clearly distinguishable constructs (manifest correlation: *r* = .54; latent correlation: *r* = .71). The relation was weaker for female than for male students, which could not be explained by measurement differences between the two groups. There were several students with high representational competence and low conceptual knowledge, but only few students with low representational competence and high conceptual knowledge.

**Conclusions:**

These results support the assumption that representational competence is a prerequisite, yet insufficient condition for the acquisition of conceptual knowledge. We provide suggestions for supporting learners in building representational competence, and particularly female learners in utilizing their representational competence to build conceptual knowledge.

**Supplementary Information:**

The online version contains supplementary material available at 10.1186/s40594-023-00435-6.

In science education, multiple external representations such as texts, graphs, charts, or formulae are commonly used to support learners’ acquisition of conceptual knowledge (Ainsworth, 2008; Corradi et al., 2012; Treagust et al., 2017). These different forms of representations provide learners with specific information about the learning object. However, to understand, apply and transfer these different forms, learners need a set of skills that is collectively referred to as *representational competence*. Kozma and Russel (2005) define representational competence as the ability to interpret, generate, and switch between different forms of representation.

It is commonly assumed that without sufficient representational competence, learners will struggle to build knowledge about the represented concepts (e.g., Kohl et al., 2007). This assumption predicts a considerable relation between conceptual knowledge and representational competence. This relation has rarely been examined (Stieff & DeSutter, 2021). The few available studies that attest to a positive association between the two constructs (e.g., Nieminen et al., 2013) did not focus on this relation, did not use psychometrically validated assessment-instruments, and applied measures of representational competence that were likely confounded with conceptual knowledge (Nieminen et al., 2013; Nitz et al., 2014a, 2014b). Prior studies also left out possible differential effects that might help explain why females tend to struggle more with acquiring conceptual knowledge in STEM (i.e., science, technology, engineering, and mathematics) than males (e.g., Liu et al., 2008; Madsen et al., 2013).

In the present study, we attempt to close these research gaps. Using psychometric measures of representational competence with fields and conceptual knowledge about electromagnetism, a central topic in physics education in which fields play a crucial role, we examine the relation of students' representational competence and conceptual knowledge. We study this relation in undergraduate students, considering differences between females and males.

## Introduction

### Representational competence in science learning

A central learning goal in science education is to improve students’ conceptual knowledge, that is, relational knowledge about the concepts within a domain (Goldwater & Schalk, 2016). For example, in biology, learners are expected to acquire conceptual knowledge about photosynthesis, in chemistry about ionic, covalent, and metallic bonds, and in physics about electromagnetism. To this end, science education commonly includes the extensive use of different types of external representations that depict or describe specific features of scientific phenomena or concepts (Treagust et al., 2017). Disciplinary representations, such as line graphs, reaction equations, or circuit schematics, for example, are considered tools for students' conceptual development, concept-related reasoning, problem solving, and scientific communication (e.g., Hubber et al., 2010; Rau, 2017).

The use of representations can place considerable challenges on learners’ representational competence (Kozma & Russel, 2005). Representational competence, which enables learners to interpret generate, and switch between different representations, is considered a prerequisite for successful conceptual learning (e.g., Kohl et al., 2007; Nitz et al., 2014a, 2014b; Nitz et al., 2014a, 2014b; Scheid et al., 2018). As Medina and Suthers (2013) put it, since conceptual knowledge builds with representational activity such as deliberately using multiple representations for learning or communicating about science concepts, representational competence is a constituent of one’s conceptual knowledge. Similarly, Scheid et al. (2018) argue that scientific representations are often *domain-specific*, implying that representational competence and conceptual knowledge might be inherently related.

*Meta-representational competence* means dealing with representations at a particularly high level, for example, by creating new forms of representations for creative problem solving. Such activities are considered particularly conducive for conceptual learning across domains (diSessa, 2004; Sherin, 2000).

Whereas these assumptions sound theoretically compelling, little empirical research has been conducted to pin down the quantitative relation between representational competence and conceptual knowledge. In addition, the available research struggles with methodological issues.

In an intervention study in the context of biology, Nitz et al., ([Bibr CR76][Bibr CR77]) found a positive correlation between secondary school students’ representational competence regarding visual-graphical and symbolic representations, and conceptual knowledge about photosynthesis. In addition, both constructs moderately predicted each other’s development from before to after a teaching sequence. Nieminen et al. (2013) found a strong positive correlation of upper secondary school students’ representational competence regarding visuo-spatial and symbolic representations with conceptual knowledge, both embedded in the context of Newtonian mechanics in the Force Concept Inventory (FCI; Hestenes et al., 1992). In addition, representational competence exhibited moderate predictive value for students’ learning gains after a course on kinematics, force, and Newton’s laws encompassing nine lessons (Nieminen et al., 2013). Scheid et al. (2019) found that secondary school students’ conceptual knowledge in optics predicted their representational competence regarding textual, diagrammatic, and formal representations within this topic, as well as improvement therein after an intervention. Going further into the mutual interplay between the two constructs, it has been found that in introductory organic chemistry, students’ prior knowledge (including both factual and conceptual knowledge) predicted whether and how efficiently they adopted new visual representations (Hinze et al., 2013). In contrast, a recent study indicated little relation of the abilities to construct and select representations with conceptual knowledge, although based on a very small sample (Stieff & DeSutter, 2021).

### The assessment of representational competence

The outlined studies providing evidence on the relation between representational competence and conceptual knowledge have at least two limitations. First, they did not focus on quantifying this relation. Instead, these studies focused on examining the dynamic interplay of representational competence and conceptual knowledge within specific learning contexts (Nieminen et al., 2013; Nitz et al., 2014a, 2014b; Scheid et al., 2019; Stieff & DeSutter, 2021). For this reason, these studies did not employ psychometrically developed assessments of representational competence (e.g., Klein et al., 2017; Scheid et al., 2018). As we will argue, psychometrically developed instruments allow controlling important factors related to measurement quality.

Second, these studies assessed learners’ representational competence and their conceptual knowledge within similar topical contexts (Nieminen et al., 2013; Nitz et al., 2014a, 2014b; Scheid et al., 2019). This is also the case for available psychometrically validated measures of representational competence, which also follow the practice of embedding the assessment within disciplinary contexts (Klein et al., 2017; Scheid et al., 2018). Whereas for some research purposes, it is useful to embed measures within typical learning contexts, for the purpose of pinning down the exact relation between representational competence and conceptual knowledge, this practice induces a methodological artefact. Specifically, embedding the items related to both constructs within the same or similar topical contexts induces confounding. Such confounding likely leads to an overestimation of the relation between the two constructs. Indeed, prior studies embedding measures of both constructs within the same topical contexts have yielded rather strong estimates of their relation (Nieminen et al., 2013; Nitz et al., 2014a, 2014b; Scheid et al., 2019). Dissimilar contexts on the other hand might deflate the relation. For example, a recent study found little relation between the same aspects of representational competence across two dissimilar topics, indicating that the variance that is captured by measures of representational competence depends significantly on the context (Chang, 2018).

For the present study, we utilize a measure of representational competence that does not explicate a specific topical context. Our employed test of representational competence is a psychometrically validated measure that involves different types of representations of vector fields (Küchemann et al., 2021). Such field representations are essential across many contexts in physics. For example, they are used to describe electric and magnetic fields, gravitational fields, and velocity fields of fluid flows. Students are usually first confronted with vector fields in physics courses in the context of electromagnetism, which we chose as topic for our employed test of conceptual knowledge. The test of representational competence presents vector fields without utilizing the topical context of electromagnetism. This instrument thus allows examining the relation between representational competence and conceptual knowledge without relying on a confounding topical context. This measure assesses two components of representational competence: Understanding how information is encoded in one form of representation, and translating from one form of representation to the other. Other components of representational competence that require reference to a specific context (e.g., meta-representational competence) are not measured. We employ this measure to examine how representational competence regarding fields relates to individuals’ conceptual knowledge about electromagnetism.

### Representational competence in electromagnetism

**Fig. 1:**
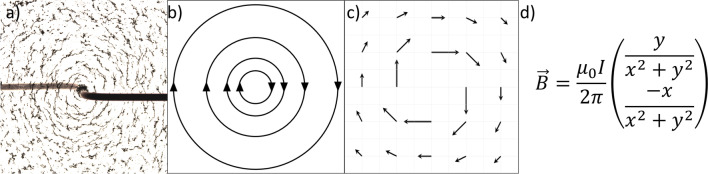
Different types of representations of the magnetic field around a current-carrying conductor. **a** shows a concrete-analog representation, **b** field lines, **c** vector-field plot, and **d** mathematical-symbolical representation

Electromagnetism is a standard topic in national and international high-school physics curricula (ISB, 2012; National Research Council, 2012). Due to its abstract nature, it is typically considered a very challenging topic for learners. Two central concepts within this topic are those of electric and magnetic fields. Electric and magnetic fields are vector fields, which means that they contain information about the direction and the magnitude at each point in space. Apart from mathematical-symbolical equations and concrete-analog illustrations in demonstration experiments (e.g., with iron filings), electric and magnetic fields are typically visualized via two types of representations, namely vector-field plots and field lines. In general, vector-field plots and field lines are convention-based representations that can be embedded in a certain context, such magnetic or electric fields, or they can be context-independent, for instance, to visualize mathematical functions of vector fields. Examples of different representations of fields are provided in Fig. 1. Each of these representations comes with certain affordances for learners and is linked to specific difficulties (Bollen et al., 2017; Küchemann et al., 2021). For instance, students often confuse the conventions of how vector-field plots and field lines depict the magnitude, which is indicated by the length of the arrows and the density of the field lines, respectively. Other difficulties of students are related to the identification of the direction of a vector field and the translation between vector-field plots and field line representations, specifically the understanding that vectors in vector-field plot are tangents to the field lines (Küchemann et al., 2021).

When applying these representations in the context of electric and magnetic fields, further difficulties can arise in learners. Albe et al. (2001) observed that many “students did not make a link between the physical concept that makes up the magnetic field and its representational modes” (p. 202). For example, even students who were aware that the magnetic field is a vector quantity did not use vectors to superpose two fields or to draw a field line representation of a uniform magnetic field. Furthermore, Sağlam and Millar (2006) found that some students think of magnetic fields as a “flow” of something. Consequently, they think that charged particles experience forces along the field, which, however, is a misinterpretation of the field lines representing the magnetic field.

Representational difficulties might also be related to students’ conceptual difficulties with electric and magnetic fields (e.g., Bagno & Eylon, 1997; Ding et al., 2006; Maloney et al., 2001). Students’ understanding of these topics tends to be fragmentary and not well-integrated into a coherent framework (Sağlam & Millar, 2006). Typical student difficulties concern confusions between electric and magnetic fields. For instance, some students believe that the field around a magnet suddenly ends and does not have an infinite extension, and that the magnetic field always points radially away from the magnet (Ding et al., 2006). To sum up, electromagnetism is an important and basic, yet very challenging topic. Learning difficulties might be related to the abstract concepts to be acquired, but also to the multiple external representations that play a central role for this topic. Electromagnetism therefore seems to be a suitable context for investigating the relation between representational competence and conceptual knowledge.

### Gender differences in representational competence

Research commonly finds lower conceptual knowledge in females than in males across various physics topics (e.g., Hofer et al., 2018; Madsen et al., 2013; OECD, 2009). Extensively researched reasons for the gender gap in some STEM fields such as physics and computer science (Cheryan et al., 2017) encompass gender differences in interest, motivation, self-concept (Jansen et al., 2014; Kang et al., 2019; Patall et al., 2018), and visual-spatial abilities (Reilly et al., 2017; Reinhold et al., 2020; Yoon & Mann, 2017), or a lack of female role models (Chen et al., 2020; Mullis et al., 2016). In the present study, we do not focus on affective-motivational factors, basic cognitive abilities, or learners’ schooling environment. Instead, we address representational competence, which is a factor of particular relevance to the STEM domain itself that might contribute to gender differences in conceptual knowledge.

Although not in all STEM-related topics gender differences are robustly found, in many physics topics male learners show an advantage in content knowledge compared to female learners (e.g., Hofer et al., 2018; Liu et al., 2008; Madsen et al., 2013; OECD, 2009). In comparison to these differences in content knowledge, gender differences in the use of different types of representation and in representational competence have hardly been studied. The available research indicates that female students are less able to deal with some types of representations than their male classmates and are less likely to use them for problem solving. In particular, this is the case with visual graphical representations, which make up a large part of the representations used in the STEM domain (e.g., Chan & Wong, 2019; Hegarty & Kriz, 2008; Tam et al., 2019). Lowrie and Diezmann (2011) demonstrated that boys outperformed girls in different types of mathematical-graphical tasks (e.g., axis tasks) that required the students to infer and consider spatial orientation or direction. These results are possibly associated with male advantages in spatial abilities (e.g., Reinhold et al., 2020), whose link with success in STEM fields has been demonstrated (e.g., Buckley et al., 2018). Heo and Toomey (2020) found that in undergraduates, effects of gender on learning from multimedia instruction involving visual-graphic representations were largely explained by differences in spatial abilities. In physics, Hake (2002) showed that spatial abilities exhibited a higher correlation with conceptual learning for male than for female students. Nieminen et al. (2013) found that the performance of female secondary school students in a force concept test was more dependent on the representational format in which tasks were presented than the performance of their male counterparts. This was shown by the fact that girls could not recognize that multiple isomorphic representations represented the same facts. This finding might be aligned with the assumption that males may have an advantage on tests that require mapping relations in working memory (Halpern, 2004). These less beneficial preconditions in cognitive abilities, which are required to build representational competence and abstract from different representations, might contribute to gender differences in conceptual knowledge.

A methodological problem that can arise when studying gender differences is a lack of measurement fairness. In psychometric terms, it can happen that some or all items work non-measurement-invariant across genders, which implies for example that they are more difficult to solve for one gender even when members of all genders are at the same level of the underlying latent trait (Wicherts et al., 2005). In research on conceptual understanding in physics, measurement differences between females and males have been found on the well-known Force Concept Inventory (e.g., Dietz et al., 2012; Madsen et al., 2013; Osborn Popp et al., 2011; Traxler et al., 2018). Traxler et al. (2018), for example, found some items to be more difficult for females than for males, which could explain a good part of gender differences on this measure. There are two ways to deal with a potential lack of measurement invariance. First, invariance can be statistically modeled and tested (Hofer et al., 2017; Wicherts et al., 2005). Thus, psychometric investigation of measurement invariance allows testing to which degree an instrument established a common scale across groups such as genders (Hofer et al., 2017). Second, latent variable modeling can be used to incorporate deviations from measurement invariance. This approach allows unbiased comparisons across genders despite moderate deviations (Wicherts et al., 2005). Consequently, the application of well-developed psychometric measures in combination with modeling of measurement invariance contributes to reliable and valid testing of gender differences. In the present study, in order to ensure that commonalities or differences found between genders are not mere methodological artefacts (e.g., Dietz et al., 2012), we examine measurement invariance in both assessment instruments and correct for potential violations thereof.

### Present study

In the present study, we investigate the relation between representational competence regarding vector fields and conceptual knowledge about electromagnetism in female and male undergraduate students. The assessed students, from universities in Germany and Switzerland, should all have received instruction about electromagnetism in their high school science lessons, but they have not yet received any further instruction on this topic at their universities. Within this context, we examine the following research questions:

(1) What is the relation between undergraduates’ representational competence regarding vector fields, and their conceptual knowledge about electromagnetism?

Based on the assumption that representational competence acts as a prerequisite, yet insufficient condition for the acquisition of conceptual knowledge (Kohl et al., 2007; Nitz et al., 2014a, 2014b; Nitz et al., 2014a, 2014b; Scheid et al., 2018), we expect a positive but imperfect correlation (i.e., *r* < 1) between the two constructs.

(2) Does the relation between representational competence and conceptual knowledge differ between female and male students?

Given the prior findings that female students typically exhibit lower conceptual knowledge in physics than male students (e.g., Hofer et al., 2018) and might also possess lower competence regarding some representations (e.g., Heo & Toomey, 2020), we examine whether the relation between the two constructs is similar or differs between these two groups of students.

## Methods

### Sample and procedure

Undergraduate students were selected as study participants, who had not yet had any experience with vector-field representations and contents in electromagnetism during their studies at university. Consequently, it can be assumed that the students were at similar levels concerning representational competence and conceptual knowledge in electromagnetism as immediately after graduating from secondary school. To gather an apt sample size, the students were recruited from different fields of study of which some were more strongly related to physics than others. It was assumed that this procedure would result in a total sample of students who had physics lessons of varying length and depth during high school. The sample size was determined by the sizes of the student cohorts at the participating universities.

The participating students received links to an online survey. Of over 1000 invited participants, 845 opened the link, and 540 completed the survey. Of those who had completed the survey, we excluded participants who had finished it in less than 10 min or had indicated on a validation question that their data was not trustworthy. The final sample consisted of *N* = 515 participants. The students came from universities in Germany and German-speaking Switzerland. They were recruited within four different courses, with the first course consisting of teacher education students in STEM and non-STEM fields (*n* = 188, 71 female, *M*_age_ = 20.75, *SD* = 3.82; an average of 4.45 years of physics at school). In the second course, students came from mechanical engineering and electrical engineering (*n* = 149, 14 female/1 diverse or unspecified, *M*_age_ = 20.72, *SD* = 2.36; average 5.57 years of school-physics). The third course was offered for students from environmental sciences (*n* = 98, 69 females/3 diverse or unspecified, *M*_age_ = 21.10, *SD* = 1.60; average 3.11 years of school-physics), and the fourth course was offered for physics students (*n* = 80, 29 female/3 diverse or unspecified, *M*_age_ = 19.74, *SD* = 2.96; average 4.08 years of school-physics). Overall, the majority of our participants were undergraduates specialized in STEM who had not yet had university education in electromagnetism. For further details on the sampling procedure, see Küchemann et al. (2021).

The students were provided with a link to the survey and could participate within their regular lecture hours or within two weeks after. In addition to the tests on representational competence and conceptual knowledge in electromagnetism, demographic and school background information as well as some information on students’ attitudes towards science (which is not part of the present analysis) was assessed. Information on all assessed variables is provided in Malone et al. (2021). The average time required to fill out the survey was 25 min. Participants provided informed consent for data usage. The study was conducted in full accordance with the ethical standards for research of the American Psychological Association’s “Ethical Principles of Psychologists and Code of Conduct” (American Psychological Association, 2017). The first author’s Swiss institution as well as German regulations did not require formal ethical approval for studies obtaining anonymized data on adult students within university courses.

### Transparency and openness

We report how we determined our sample size, all data exclusions, all manipulations, and all measures in the study. All data and research materials are available from https://osf.io/p476u, Küchemann et al. (2021), and Malone et al. (2021), and the analytic scripts for the present analysis from https://osf.io/rfyh6/. Data were analyzed using Mplus, version 8.6 (Muthén, & Muthén, 1998-2021) run from within the R software environment version 4.0.2 (R Core Team, 2021) via the MplusAutomation package (Hallquist & Wiley, 2018). The *tidyverse* packages were used for data wrangling and visualization (Wickham et al., 2019). This study’s design and its analysis were not pre-registered. For statistical tests in analysis that were undertaken on the whole sample, we use 95% significance levels and report 90% confidence intervals to convey uncertainty in parameter estimates (Schweder & Hjort, 2016). For statistical tests on sub-samples (e.g., within or comparing genders), we use 90% significance levels to prevent increased rates of beta-errors, again reporting 90% confidence intervals.

## Measures

### Representational competence

**Fig. 2:**
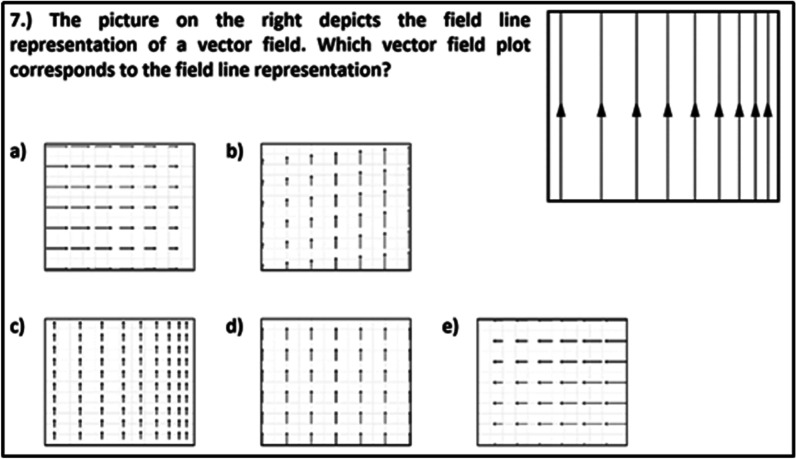
Example item from the representational competence with fields-inventory (Küchemann et al., 2021)

The inventory for representational competence of fields (RCFI; Küchemann et al., 2021) was used to assess students’ understanding of vector-field plots (4 items) and field-line representations (4 items), as well as translation between these (4 items). Ten of these items have a single-choice format and contain between 4 and 5 answer options of which always one is correct. The two remaining items have a multiple true–false format, which were considered as correct if the students chose all three respective correct answer options. For more detailed descriptions of the instrument as well as results from Rasch scaling and cognitive interviews, see Küchemann et al. (2021). The items do not explicate a specific topical context such as electromagnetism. An example item depicting how this topical context-independence is achieved is presented in Fig. 2.

To examine the psychometric validity of the instrument, a unidimensional confirmatory factor analysis was fitted using the WLSMV-estimator in the software package Mplus 8.6 (Muthén & Muthén, 1998-2021). For all latent variable models, we inspected multiple fit indices and particularly residual associations to judge the severity of misfit (Greiff & Heene, 2017). There were no outstanding residual associations in any of our models that would point towards substantial model misspecification. Including one residual covariance between two items with the same item stem, the analysis showed acceptable fit, χ^2^_53_ = 88.45, *p* = 0.002, *RMSEA* = 0.036, CI90[0.022; 0.049], *CFI* = 0.939, *TLI* = 0.923, with standardized factor loadings between 0.34 and 0.85 and no salient residual covariances. The internal consistency-estimates were α = 0.86 and ω = 0.86 (for a description of the model-based Omega-coefficient, see Dunn et al., 2014). For descriptive analyses, the sum of students’ correct answers on the instrument was used, ranging from 0 to 12 points.

Since we wanted to compare the relation between representational competence and conceptual knowledge between females and males, we examined measurement invariance across these groups to ensure that potential group differences could not be attributed to measurement bias. We followed the steps outlined by Svetina et al. (2020) that are appropriate for testing invariance of measures with categorical items. Note that these steps differ in order and details of implementation from measurement invariance analysis with continuous items; we refer readers interested in the details of the applied steps to Svetina et al. (2020). We followed their procedure to examine invariance of structure, loadings, and thresholds, which are the appropriate steps to ensure that differences in latent means or in the latent relation between the two constructs would not be a measurement artefact. Following the steps described by Svetina et al. (2020), the instrument exhibited partial measurement invariance of loadings and thresholds across females and males, with a fit of χ^2^_108_ = 158.43, *p* = 0.001, *RMSEA* = 0.043, CI90[0.028; 0.057], *CFI* = 0.970, *TLI* = 0.964. Only two factor loadings exhibited lack of measurement invariance, which we could easily accommodate and correct for in all further latent variable models to ensure fair comparisons (Van de Schoot et al., 2012). This level of measurement invariance thus allows the unbiased comparison of latent correlations and mean values across genders. Internal consistency estimates were α = 0.83, ω = 0.84 for females, and α = 0.87, ω = 0.87 for males.

### Conceptual knowledge

**Fig. 3:**
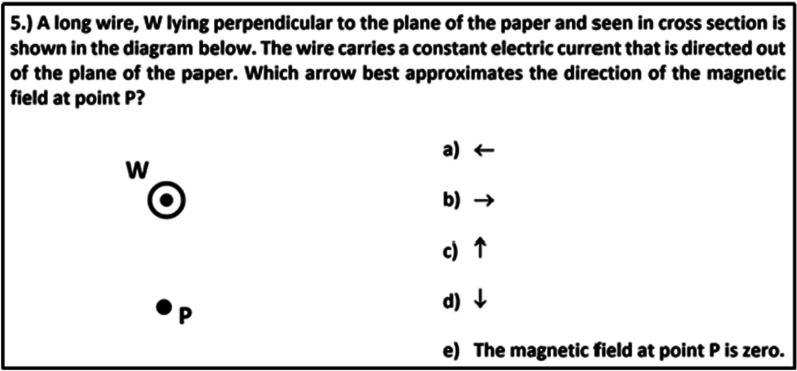
Example item from the assessment of conceptual knowledge in electromagnetism (taken from McColgan et al., 2017)

To assess students’ conceptual knowledge about electromagnetism, we compiled a test cpnsisting of 13 single-choice items with between five and ten distractors. The test covers four of the conceptual areas on electromagnetism suggested by Maloney et al. (2001) and McColgan et al. (2017), namely magnetic fields generated by magnets and electric currents, and magnetic force (also known as Lorentz force) on current-carrying wires and moving charged particles. Nine test items were adopted from established inventories by Ding et al. (2006), Maloney et al. (2001), and McColgan et al. (2017). Four additional items were developed and validated by four experts: two experienced physics professors, and two researchers with PhDs in physics education and teacher's qualifications. The test comprises three items that ask about the direction of a magnetic field at a selected point, and three items in which the magnetic field of a configuration of magnets or currents has to be determined. In three cases, the solution involves superposing magnetic fields. Considering the magnetic force, three items cover the magnitude of the force, and four items are related to the direction of the force. Determining magnetic forces requires students to relate magnetic fields to the direction of the flow of charged particles (either in a wire or freely moving). Therefore, all items include the interpretation of magnetic fields, which are either represented by single vectors of the vector-field plot (five items) or by field lines (eight items). An example item is shown in Fig. 3. In this item, students have to determine the direction of the magnetic field.

After estimation of a unidimensional confirmatory factor analysis, one item was removed that showed a very low factor loading. The item was among the more difficult items but still showed sufficient variation (27% solution rate). A potential explanation for the item’s misfit was that it demanded knowledge about a rather specific detail of the magnetic force, namely that the force affects only those parts of the conductor that are located within the range of the magnetic field. Students with generally low conceptual knowledge about electromagnetism might have learned this fact, whereas it might not have been part of instruction for those with otherwise good conceptual understanding. This item might thus cover knowledge of a specific isolated fact more than thorough conceptual knowledge, indicating a lack of validity.

After removal of this item, the unidimensional model showed acceptable fit, χ^2^_54_ = 102.78, *p* < 0.001, *RMSEA* = 0.042, CI90[0.029; 0.054], *CFI* = 0.967, *TLI* = 0.959, with standardized loadings between 0.59 and 0.84 and no salient residual covariances. Item mean values (percent solved) ranged from 0.20 to 0.47, indicating rather high item difficulties within this sample. The internal consistency estimates for the instrument were α = 0.91 and ω = 0.92. For descriptive analyses, the sum of students’ correct answers on the instrument was used, ranging from 0 to 12 points. Note that based on the number of items and answer options within each item, the expected score for an individual engaging in random guessing on this instrument would be 1.7 points.

Following the steps outlined by Svetina et al. (2020), this instrument exhibited partial measurement invariance of loadings and thresholds across females and males, with only one loading differing between genders and a fit of χ^2^108 = 230.45, *p* < 0.001, *RMSEA* = 0.067, *CFI* = 0.945, *TLI* = 0.932. Internal consistency estimates were α = 0.83, ω = 0.83 for females, and α = 0.93, ω = 0.93 for males.

### Analytic approach

We will employ the same set of analytic approaches for our two research questions. The first research question is concerned with the relation between representational competence and conceptual knowledge. In order to examine this relation, we will use three statistical tools. First, we will use a scatter plot as a visual representation of the two constructs’ relation. A scatter plot can reveal details about such a relation that might remain hidden in descriptive or inferential statistical estimates, such as the specific nature of a relation (e.g., linear, quadratic, or more complex), width of variances across the whole spectrum of the variables, and specific details of the bivariate distribution between the constructs such as learners being high only on one construct, but low on the other.

The second statistical tool we use is a linear correlation estimate with confidence interval. The reason to report such a basic statistical estimate is that in contrast to more elaborate models, such as structural equation modeling, such a statistic does not make rely on meta-theoretical assumptions such as reflective latent variables (see e.g., Borsboom, 2008; Edelsbrunner, 2022). This statistic is also commonly used in meta-analyses (e.g., Schneider et al., 2017, 2018).

Note that although we are not interested in examining similarities and differences in the relation between the constructs across the different sub-samples that were used in this study, we will correct standard errors and confidence intervals for all statistical estimates, including estimates of correlations and Cohen’s *d*s, for cluster dependence via cluster-robust maximum likelihood estimation (Szpiro et al., 2010). Comparative analyses across the four samples are presented elsewhere (Edelsbrunner & Hofer, 2023) and the data for further comparisons are freely available (Malone et al., 2021). Standard errors and confidence intervals will also be corrected for deviation from bivariate normality with a multivariate kurtosis-robust estimator (Yuan et al., 2004).

The third statistical tool that we will use is latent variable modeling (Beaujean, 2014). In contrast to manifest variables, in latent variable modeling specific meta-theoretical assumptions (e.g., that all non-shared variation between indicator variables is measurement error; Edelsbrunner, 2022; White et al., 2022) allow separating measurement error from true construct variance (Beaujean, 2014). We will use this approach to estimate the latent (i.e., measurement error-free) correlation between the two constructs. In this step, we will also test for a quadratic relation that was indicated by the scatter plot.

Finally, to examine our second research question, which is concerned with gender differences in the relation between the two constructs, we will again conduct the same three analytic steps, but separate for genders and with the following adaptations. In reporting the linear correlation between the two constructs, we will add a robustness-check across samples since the gender distribution was skewed in some of the samples. This robustness-check can be found in the Additional file 1: Table S4. We will also report estimates of covariances in addition to correlations, since differences in correlations do not necessarily have to go hand-in-hand with differences in covariances if variances of the involved variables differ between genders (Little, 2013). Both, covariances and correlations, indicate the strength of the relation between the two constructs, but from different (i.e., raw vs. standardized) perspectives. Raw regression weights will also be reported as the basis for extended interpretations of gender differences in the discussion. The third analysis, latent variable modeling, will be extended into a multigroup-model (Beaujean, 2014) to examine similarities and differences in the two latent variables across genders.

## Results

Descriptive statistics on the two main variables (conceptual knowledge and representational competence) are presented first, followed by the analyses concerning the two research questions.

### Descriptive statistics

**Fig. 4:**
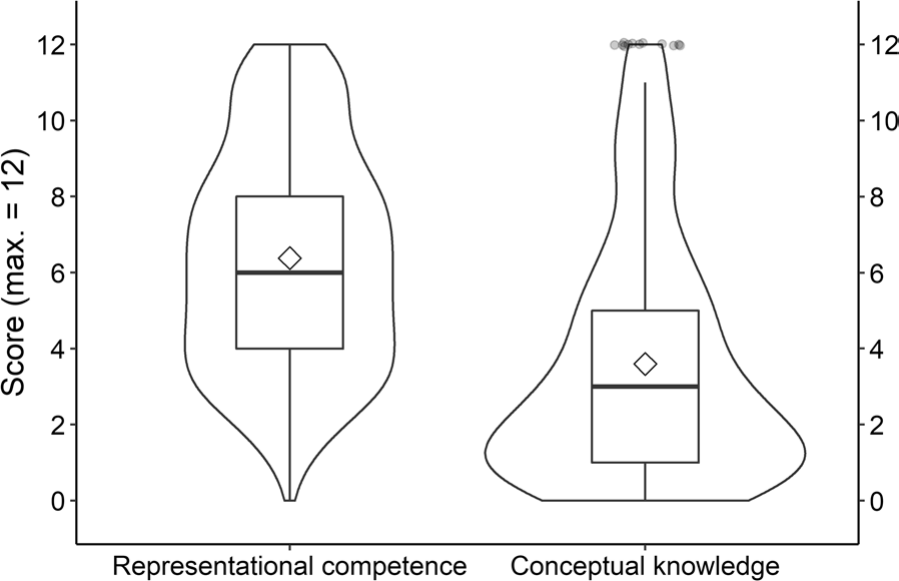
Distributions of Students’ Scores on Representational Competence and Conceptual Knowledge. Distributions are indicated as boxplots, complemented by violin-shapes outlining kernel densities and overall means indicated by diamonds. Outliers (more than 1.5 interquartile-ranges above third quartile) indicated by individual points

Figure 4 depicts distributions of students’ scores on representational competence and on conceptual knowledge. On representational competence, the students yielded a mean score of *M* = 6.38 (53.17% solved) out of 12 items, with a standard deviation of *SD* = 3.90 and range of 0–12. On conceptual knowledge, the students yielded a mean score of *M* = 3.60 (30.00% solved) out of 12 items, with a standard deviation of *SD* = 3.19 and a range of 0–12. For more detailed descriptive statistics, see Additional file 1: Table S1. Additional file 1: Table S2 presents item statistics for the representational competence-test, and Additional file 1: Table S3 for the conceptual knowledge-test. Overall, the students achieved medium scores on representational competence, and rather low scores on conceptual knowledge, which exhibited a moderate floor effect.

### Relation between representational competence and conceptual knowledge

**Fig. 5:**
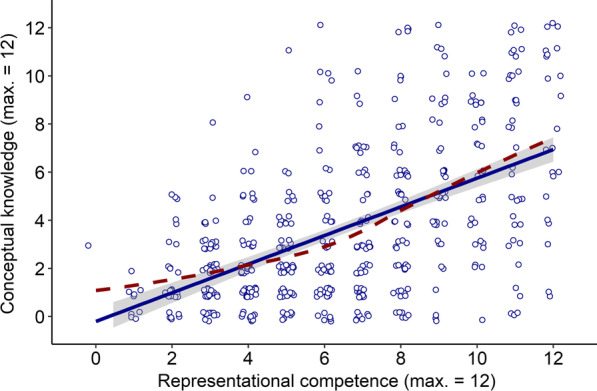
Scatter Plot Depicting Relation Between Representational Competence and Conceptual Knowledge. Points jittered for better readability. Full line indicates linear fit with 90% confidence band, dashed line smooth fit from general additive model

In order to examine the relation between students’ representational competence and conceptual knowledge, we inspected a scatter plot, estimated a Pearson correlation, and set up a latent variable model to obtain an estimate of the latent (measurement-error free) correlation between the two constructs. The scatter plot is provided in Fig. 5.

As visible from Fig. 5, there appeared to be a positive relation between representational competence and conceptual knowledge. The more representational competence students had, the more conceptual knowledge they tended to have as well. The estimated linear correlation between the two measures was significant and moderate to strong, *r* = 0.54, *p* < 0.001, with a 90% confidence interval of CI90[0.48; 0.60]. The smooth fit line in the scatter plot (dashed line in Fig. 4; stemming from a general additive model implemented via the ggplot2-package, Wickham et al., 2019) indicated that the relation between the two constructs might have a quadratic characteristic, being stronger at higher levels of the two variables. We first remain with modeling a linear relation between the two constructs and then present an explorative model including a quadratic term.

In the next step, we set up latent variable models to yield measurement error-corrected estimates of the relation between the two constructs. This model allowed testing our priorly stated hypothesis regarding this relation, namely, that it would be strong but clearly below unity (r < 1). To test this assumption, we set up the following two models. The first model was a unidimensional model, in which representational competence and conceptual knowledge were described by the same factor. This unidimensional model is statistically equivalent to a model with two latent variables that share a perfect correlation of *r* = 1. We set up this model and compared it with a second model in which the two constructs represented two separate, yet correlated latent variables. By comparing the fit of both models, we could test our hypothesis. To account for the moderate floor effect in conceptual knowledge and for cluster-dependence stemming from the four different samples, we used a cluster- and kurtosis-robust Huber-White sandwich-maximum likelihood estimator (cluster-robust MLR; Szpiro et al., 2010; Yuan et al., 2004).

The fit of the first, unidimensional assuming one latent variable to describe the common variance across all items of the two constructs, was χ^2^_251_ = 344.96, *p* < 0.001, *RMSEA* = 0.027, CI90[0.020; 0.034], *CFI* = 0.945, *TLI* = 0.939. The second model, in which the two constructs were represented by two correlated latent variables, showed a fit of χ^2^_250_ = 302.69, *p* = 0.013, *RMSEA* = 0.020. CI90[0.010; 0.028], *CFI* = 0.969, *TLI* = 0.966. The fit indices of the second model with two correlated latent variables appeared better. To statistically test this impression, we conducted a chi-square model difference test (Asparouhov & Muthen, 2010). The test supported this impression, showing that the model assuming two latent variables instead of one exhibited significantly better model fit, Δχ^2^_1_ = 43.19, *p* < 0.001. The latent linear correlation between the two constructs in the two-dimensional model was *r* = 0.71, *p* < 0.001, CI90[0.66; 0.77]. These results provide evidence that the relation between representational competence and conceptual knowledge in this sample is substantial, however far from unity (i.e., *r* < 1.00).

Since the scatter plot (Fig. 5) indicated that the relation between the two constructs might be described as quadratic, we also set up a quadratic latent variable model. We present both models but interpret the linear model as our main model and the quadratic model as an additional explorative model because the quadratic part of the relationship appears not very pronounced and adds interpretational difficulty, as a quadratic model has to be set up as a regression instead of regular correlation. Specifically, in order to be able to include a quadratic term for the relation between the two constructs, we had to predict conceptual knowledge from representational competence, including both linear and quadratic regression terms. This required maximum likelihood-estimation with numerical integration. A Satorra-Bentler scaled Chi-square difference test (Satorra & Bentler, 2010) was used to test for significance of the quadratic regression term. Model estimates confirmed a quadratic relation between the two latent variables (Δχ^2^_1_ = 20.65, *p* < 0.001), with an estimated linear regression term of *b* = 1.32, (β = 0.68), and an estimated quadratic regression term of *b* = 0.30 (β = 0.17). The positive quadratic term showed that as indicated by the scatter plot, the relation was stronger at the upper end. Overall, via the linear and quadratic regression terms, students’ representational competence could explain 52% of variance in their conceptual knowledge.

### Gender differences in the relation between representational competence and conceptual knowledge

To examine gender differences in the relation between representational competence and conceptual knowledge, we investigated descriptive statistics and scatter plots for the means, covariances, and correlations across genders, and then estimated a multiple-group latent variable model (Beaujean, 2014) in which we could compare unstandardized (i.e., covariances) and standardized (i.e., correlations) estimates of the relation across genders.

**Fig. 6:**
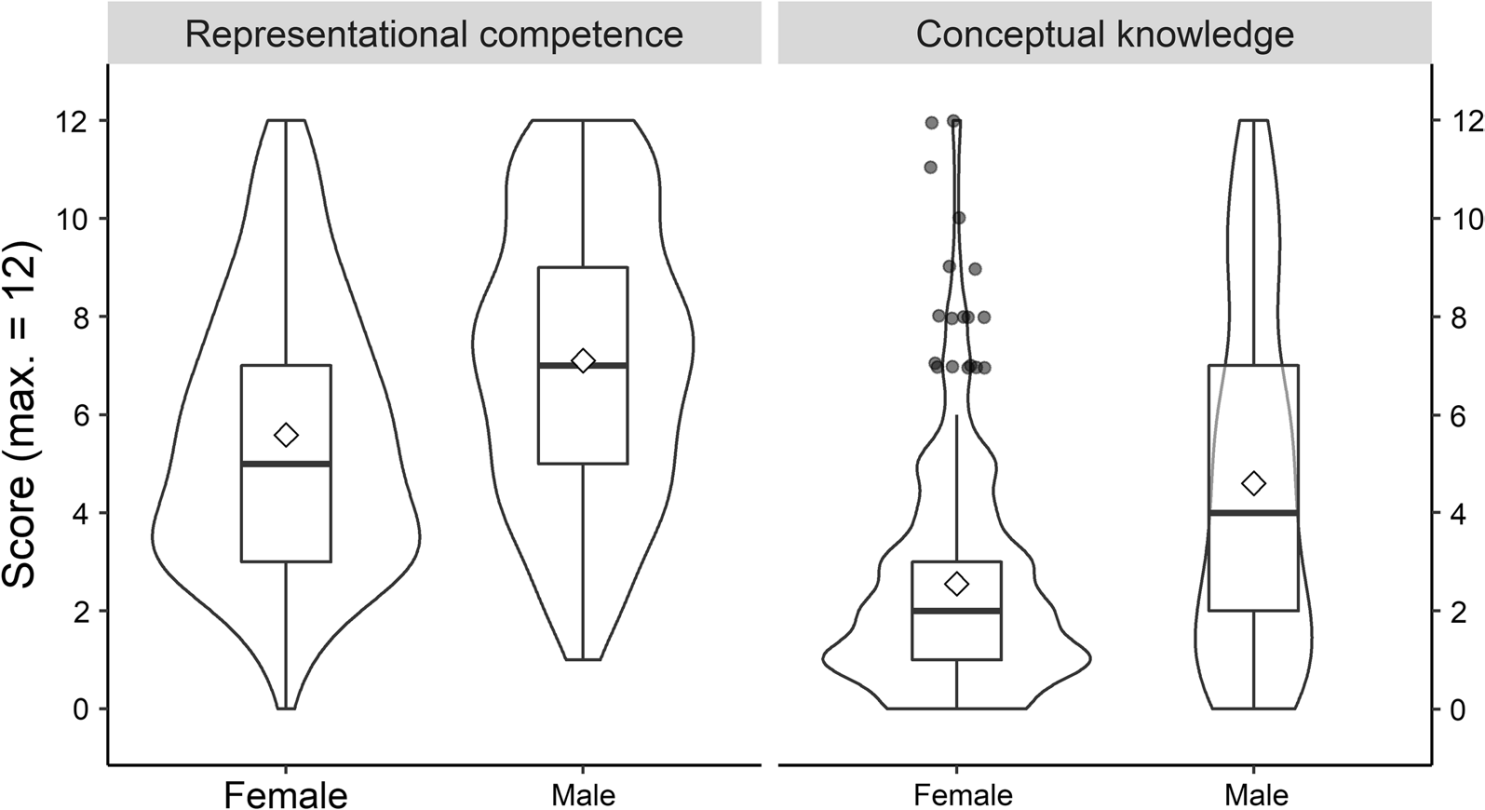
Distributions of Females’ and Males’ Scores on Representational Competence and Conceptual Knowledge**.** Distributions are indicated as boxplots, complemented by violin-shapes outlining kernel densities and overall means indicated by diamonds. Outliers (more than 1.5 interquartile-ranges above third quartile) indicated by individual points

The distributions of scores on both instruments are depicted separately for female and male students in Fig. 6. Descriptive statistics indicated that females showed a lower mean score on representational competence (*M* = 5.59, *SD* = 2.71) than males (*M* = 7.10, *SD* = 2.89), with a standardized mean difference of Cohen’s *d* = 0.55, CI90[0.40; 0.69]. Females also showed a lower mean score on conceptual knowledge (*M* = 2.55, *SD* = 2.30) than males (*M* = 4.60, *SD* = 3.58), with a standardized mean difference of Cohen’s *d* = 0.68, CI90[0.60; 0.77]. The plot in Fig. 6 corroborates these numbers, showing that on representational competence, the highest density of the distribution was around 3 points for females and around 7 points for males. On conceptual knowledge, males showed the highest density at only about 1 or 2 points, and females were even lower, representing statistical outliers when they obtained a high result (indicated by individual points in Fig. 6). It will be discussed how some of these results might represent guessing. Students indicating gender diverse identity (*n* = 7) showed mean values of *M* = 6.00, *SD* = 2.55 on representational competence, and *M* = 1.80, *SD* = 1.92 on conceptual knowledge, indicating average representational competence but low conceptual knowledge within this group. This group of students was too small to be included in the further statistical models.

**Fig. 7:**
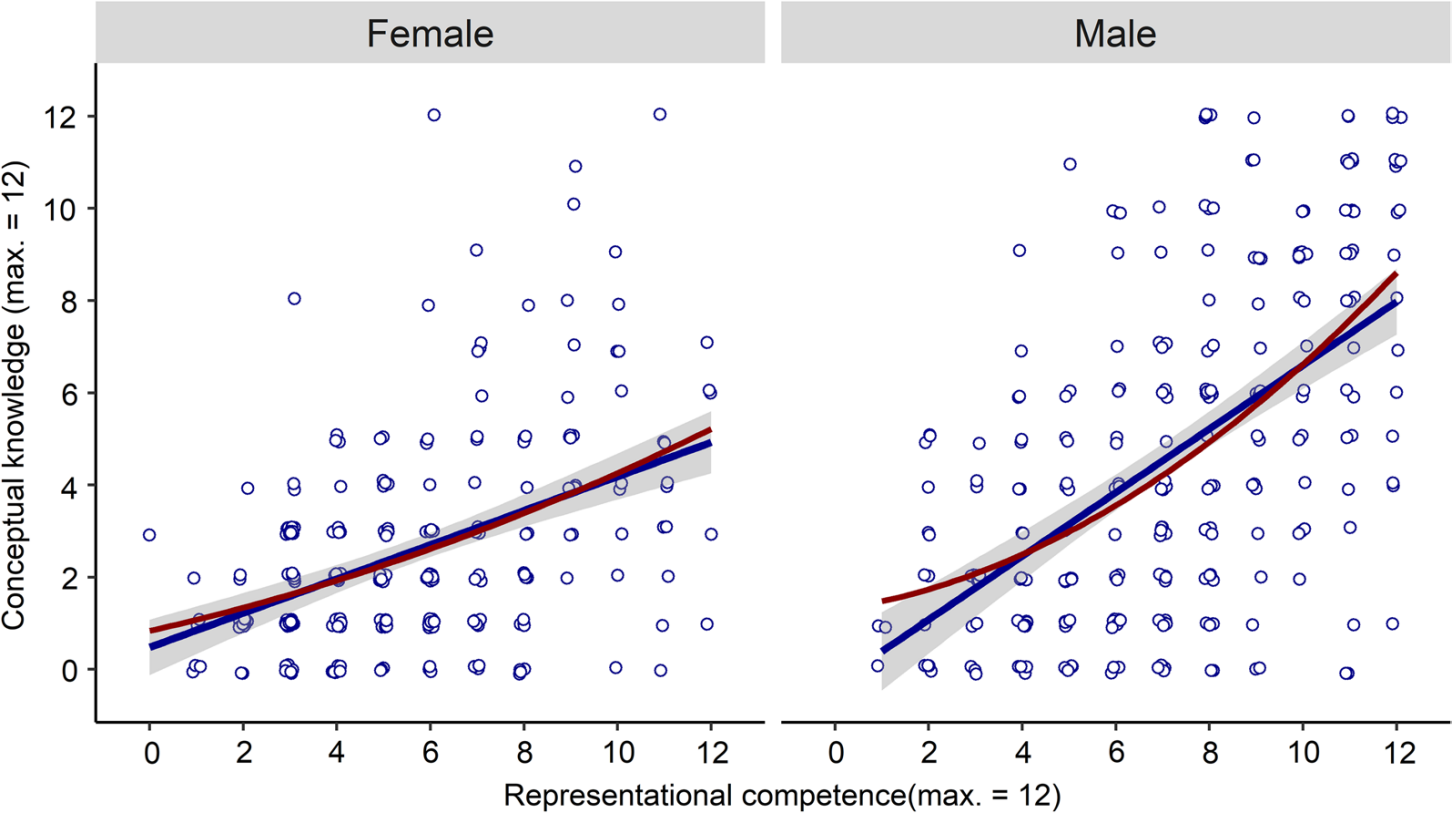
Relation Between Representational Competence and Conceptual Knowledge in Female and Male students**.** Points Jittered for Better Readability. Full lines indicate linear fit with 90% confidence bands, dashed lines quadratic fit

The estimated covariances of students' sum scores on representational competence and conceptual knowledge were *cov* = 2.92 for females, and *cov* = 5.74 for males. Note that these covariance estimates translate into regression weights of *b* = 0.37 for females, respectively* b* = 0.69 for males for predicting conceptual knowledge from representational competence. Pearson correlation estimates showed an estimated correlation between the two constructs of *r* = 0.44, *p* < 0.001, CI90[0.38; 0.50] for females, and *r* = 0.56, *p* < 0.001, CI90[0.50; 0.62] for males. In accordance with these estimates, Fig. 7 indicates a weaker association for female students than for male students. These results were relatively robust across samples (Additional file 1: Table S4).

We examined whether these differences in the relation between the two constructs could also be found on the latent level. Differences in the latent association between males and females were modeled by extending the latent variable model that we used for the first research question to a multigroup-model (Beaujean, 2014) that allowed comparing parameters between females and males. In this model, we implemented invariance of item loadings and thresholds according to the results from the measurement invariance-results described in the method section. We first remained with modeling a linear association, because the quadratic term in the overall sample was significant yet rather weak. Introducing quadratic terms into the model necessitates specification of a regression instead of correlation, as well as numerical integration in the estimation, which is computationally demanding. We therefore abstained from quadratic terms at first and remained with inspecting latent correlations between genders, before trying to fit a quadratic implementation. Within the linear model, we first examined whether the covariance estimate between representational competence and conceptual knowledge differed between males and females. In accordance with the descriptive statistics, the estimated covariance turned out to be weaker in in females, *cov* = 0.69, CI90[0.65; 0.74] than in males, *cov* = 1.65, CI90[1.05; 2.23], with a difference test corroborating a difference between these covariances, Δχ^2^_1_ = 29.79, *p* < 0.001. A different picture emerged when comparing the resulting correlation coefficients on the latent level by standardizing these covariances based on the genders’ latent variance estimates. The latent correlation coefficient for females, *r* = 0.69, CI90[0.65; 0.74], was very similar to that of males, *r* = 0.70, CI90[0.62; 0.78]. Note that the estimate for the females is equal to the females’ covariance estimate because the variances of the latent variables in that group were fixed to 1 for identification of the latent scales (Kline, 2015). The similarity in latent correlations despite differences in latent covariances can be traced back to differences in the two groups’ latent variances. The female students had a moderately lower latent variance estimate in representational competence (females: fixed to 1.00 for identification, see Kline, 2015; males: 1.25) and more strongly so in conceptual knowledge (females: fixed to 1.00; males: 4.38). Since the latent correlation coefficients are standardized based on these latent variances, the estimated correlation for females turned out so similar in contrast to the differences in manifest correlations.

We finally fitted the quadratic extension of the latent variable model across genders, to explore how similar or different females and males were in the linear and quadratic parts of their associations. Whereas the quadratic part of the relation was similar in both groups, females: *b* = 0.038, *p* = 0.006, males: *b* = 0.040, *p* < 0.001, the linear term was weaker in females, *b* = 0.17, *p* < 0.001 than in males, *b* = 0.28, *p* = 0.002.

## Discussion

In this study on undergraduates from Germany and Switzerland, we found that representational competence regarding visual-graphical field representations and conceptual knowledge about electromagnetism show a substantial relation, yet the two constructs’ relation is far from unity (i.e., *r* < 1). Results indicated a quadratic relation that was weaker in female than in male students. We discuss these results and their implications for science education and research in turn.

### The interrelation between representational competence and conceptual knowledge

The substantial positive association between representational competence and conceptual knowledge supports the argument by Scheid et al. (2018) according to which the two constructs might develop interdependently and even bootstrap one another. A positive relation between representational competence and conceptual knowledge has been found before (e.g., Nieminen et al., 2013). However, in this and further prior studies representational competence was measured in the same topical context which might inflate the relation. It is therefore noteworthy that in the present study, the two constructs exhibited a substantial relation despite being measured without confounding topical context.

As representational competence and conceptual knowledge build together, representational competence is often seen as a constituent of one’s conceptual knowledge (Medina & Suthers, 2013). However, the two constructs were found to be clearly statistically separable. There might be several reasons why the statistical relation between the two constructs was not stronger. First, the development of conceptual knowledge in STEM instruction relates to a number of aspects that might be (partially) independent of representational competence. These may include, for example, more general reasoning abilities (Stelzer et al., 2021), prior conceptions developed from everyday experiences (Edelsbrunner et al., 2018), prior knowledge from former instruction on related topics (e.g., on forces; Simonsmeier et al., 2022), and affective-motivational factors (Cordova et al., 2014).

Moreover, reference should be made to the further facets of representational competence and the concept of meta-representational competence, which relate to a person's ability to understand the meaning of representations in the respective domain and the specific concepts (diSessa, 2004; diSessa & Sherin, 2000). For groups of people with very high meta-representational competence, the correlation of the two constructs might approximate 1. In other words, individuals who understand very well how to best solve a specific conceptual task using or creating the appropriate type of representation might be able to perfectly invest their representational competence into conceptual knowledge. Note that this assumes that (meta-) representational competence is the only decisive factor for building up conceptual knowledge, which should be scrutinized theoretically and empirically. At a lower level of meta-representational competence, the two may develop more independently (although high conceptual knowledge is probably virtually impossible at low meta-representational competence). Differences in meta-representational competence could in turn be due to differences in the emphasis placed by teachers on addressing the relationship between representation and concept in their lessons through representational activity tasks (Scheid et al., 2019), or to differences in the efforts that individuals invest into relating the two.

Besides the observation that there were students with high scores in representational competence and low scores in conceptual knowledge, Fig. 4 shows that there were almost no students with the opposite pattern: High scores in conceptual knowledge did rarely occur with low representational competence. This supports the assumption that representational competence is a prerequisite for the acquisition of conceptual knowledge (e.g., Kohl et al., 2007; Nitz et al., 2014a, 2014b; Nitz et al., 2014a, 2014b; Scheid et al., 2018).

A related explorative finding is the quadratic type of relation between the two constructs. A reasonable explanation lies in the observed moderate floor effect in the scores on conceptual knowledge. If many students have very low scores in conceptual knowledge but low to medium scores in representational competence, the correlation curve naturally flattens out on the lower end (see Fig. 6). Rather low overall scores in conceptual knowledge are in line with earlier findings based on similar test items (Ding et al., 2006; Maloney et al., 2001; Sağlam & Millar, 2006). Considering the expected test guessing score of 1.7, the observed floor effect with many scores between 0–3 points might be attributed to guessing. However, the quadratic relation remained when it was estimated on the latent level, where most variance that can be attributed to guessing should be corrected for (for details on this, see e.g., Holster & Lake, 2016). This robustness indicates that the quadratic relation might be more than a statistical artefact. For example, it might suggest a threshold in representational competence necessary for building conceptual knowledge. Since our study is the first to focus on this relation in such detail, the quadratic relation needs to be replicated on further samples and with alternative analytic approaches (e.g., Weiss et al., 2020) to examine whether it holds across samples and contexts and goes beyond a statistical artefact.

From these results, we infer the hypothesis that it seems to be worthwhile for teachers to invest time in building representational competence in their students in order to support them in learning physics concepts. However, the findings also show that even a high level of representational competence does not guarantee success in understanding physics concepts.

### Differences in males’ and females’ relation between representational competence and conceptual knowledge

The weaker relation between representational competence and conceptual knowledge in females may indicate that the males in our sample had higher meta-representational competence (diSessa, 2004; diSessa & Sherin, 2000). This gender difference could result from the fact that the females might have failed to use their developing representational competence to build conceptual knowledge on electromagnetism in school. Assuming that the effect of representational competence on conceptual knowledge is causal (note that the present study examined relations, not causality, so this is a hypothetical assumption), the estimated regression coefficients indicate that each point of representational competence translates into 0.37 points of conceptual knowledge for females, and into 0.69 points for males. In these raw terms, we would infer males to do almost twice as well as females in investing their representational competence into conceptual knowledge. If one further assumes that the two abilities bootstrap each other during learning (Scheid et al., 2019), this may be one mechanism of why females are less likely than males to perform outstandingly in some science subjects (e.g., Meho, 2021): When males have acquired a certain level of representational competence, they can invest it into the advancement of their conceptual knowledge, but this process might work worse for females. Since this is a correlational study, however, we do not yet know how much of this relation is causal. In addition, a part of the weaker relation found in females might be explained by range restriction due to floor effects. At the same time, our results clearly show that even for females with high representational competence, the relation is much weaker than in males, ruling out such a statistical artifact as a sufficient explanation. We suggest running replication studies in students from university and high schools. This needs to be done to examine whether the weaker relation of representational competence and conceptual knowledge in females generalizes to further contexts and populations and might really hint at differential dynamics.

It is noteworthy that in contrast to the covariance between the two constructs, the difference in the relation appeared more moderate in the manifest correlation estimates, and the latent correlation estimates even appeared very similar between females and males. In other words, whereas the females’ covariance between the two constructs is clearly smaller than that of the males, set in relation to the much smaller variances in both constructs for females, the resulting latent correlation coefficients are very similar. This finding points to the importance of using graphical inspections and unstandardized statistical approaches in order not to overlook differences in relations that might not be apparent in standardized coefficients such as correlations, an observation that relates to discussions almost 100 years old (Wright, 1923). The manifest correlations in our sample appeared more different than the latent correlations. This can be accounted to the fact that whereas the (unstandardized) parameters in the measurement models were very similar in both groups, as evidenced by our tests of measurement invariance, the residual variances were larger in the female sample. This is reflected in the higher estimates of internal consistency according to the Alpha and Omega indices in males. The latent variable models corrected for the resulting higher amount of measurement error in females, making the latent correlations even more similar to each other than the manifest estimates. An aspect that should be considered in interpreting this result is the strong meta-theory that latent variable models imply (White et al., 2022). Latent variable theory induces strong assumptions about the modelled constructs, such as all the non-shared variance between items representing (measurement) error (Borsboom et al., 2003; Hair & Sarstedt, 2019; Kline, 2015). For constructs in education (Edelsbrunner, 2022; White et al., 2022) and more specifically for conceptual knowledge (Stadler et al., 2021; Taber, 2018), it has been recently debated whether traditional latent variable models with this specific assumption regarding measurement error are useful. We leave it to readers to decide whether they prefer interpreting the manifest, or the latent estimates of the two constructs’ covariances and correlations. In both approaches, the covariance, which is the primary statistical estimand regarding our research question of gender differences (Lundberg et al., 2021), turned out to be clearly smaller in females.

It should be noted that the gender differences reported here could be caused by many factors. While there is still no agreement about the relative importance of socio-cultural and biological factors, most researchers concur that the gender gap in science domains can be considered a product of both nature and nurture (e.g., Stewart-Williams & Halsey, 2021; Stoet & Geary, 2018). The gender differences found in this study can hence be expected to largely reflect differences in exposure and prior experiences in and out of school (Quaiser-Pohl & Lehmann, 2002). In the context of science and scientific thinking, both teachers (e.g., McCullough, 2002; Taasoobshirazi & Carr, 2008) and parents (e.g., Crowley et al., 2001; Tenenbaum & Leaper, 2003), for example, tend to put more demanding questions on, and engage in more sophisticated communication with, boys than girls.

To gain further insight into possible explanatory variables for the gender gap, future studies should additionally assess spatial ability. However, since spatial ability is a broad construct comprising several factors (Carroll, 1993), the applied spatial ability tests should be chosen carefully and matched to the spatial requirements of the tasks to draw explanatory conclusions. Moreover, although spatial ability is considered a general cognitive ability (Lohman, 1996) and males are assumed to profit from an initial advantage based on social and biological factors (Reilly et al., 2017), evidence suggests that it is highly trainable (Uttal et al., 2013a, 2013b). Previous research showed that spatial training can reduce the gender gap regarding spatial abilities (Uttal et al., 2013a, 2013b), improve grades in physics courses (Miller & Halpern, 2013) and even increase the gender diversity in professional STEM fields (Sorby et al, 2018). In the course of their educational path, males become increasingly superior with respect to their spatial intelligence due to biological and environmental factors (Baenninger & Newcombe, 1989). Since we expect representational competence to be closely related to spatial ability, early STEM education should break the widening and consolidation of the gender gap by addressing spatial learning and link it to content-related representational tasks. Tzuriel and Egozi (2010) already showed that a training program on improving representation and transformation of visuospatial information in young children could close the spatial ability gender gap. In studies taking up such training programs, it might be examined whether after undergoing such training, learners manage to improve their conceptual knowledge, and if such trainings for females can close a part of the gender gap in representational competence and conceptual knowledge.

In line with the ability-as-compensator hypothesis, assuming that spatially weak learners benefit from explicit visuo-spatial support (Hays, 1996; Höffler, 2010), Yezierski and Birk (2006) showed for conceptual understanding in chemistry that additional molecular-level animations could eradicate initial gender differences in middle-school students. Based on this, it should be investigated if the use of animations, for example illustrating Maxwell’s law, Faraday’s law, or a Hertzian dipole, as these concepts explicitly address temporal changes in magnetic and electric fields, could be used as a simple tool to support spatially weak learners’ conceptual understanding of electromagnetism.

A further step should be to explore how learners could be promoted in the classroom to combine representations and concepts in order to facilitate high achievement and access to STEM careers. To motivate them to invest more in making these essential connections, care should be taken to incorporate activities into STEM lessons that girls prefer, such as those that involve cooperative work, address real-world problems, and have creative elements (for a review, see Meece et al., 2006). In particular, this last aspect seems promising, as creating and inventing representations for concepts can foster meta-representational competence (diSessa, 2004) and conceptual knowledge acquisition (Scheid et al., 2019).

## Limitations

The present study only provides an observational look into the relation between representational competence and conceptual knowledge. A part of the observed relation in our study might be explained by unmeasured confounding variables. At the very least, however, we very likely have obtained an estimate of the upper asymptote of the relation between the two constructs that has built up through their causal interplay (Ryan & Dablander, 2022) in undergraduates. By using equilibrium causal models, cross-sectional data like ours might be used to yield a first approximation of the potential causal interplay between the two constructs (Ryan & Dablander, 2022). We suggest further examining the dynamic interplay between representational competence and conceptual knowledge in longitudinal and experimental studies. In a longitudinal design, we suggest assessing both constructs repeatedly during phases in which learners receive relevant school instruction. This could for example be done while they are working on theoretical materials about physics-topics that are accompanied by experiments employing multiple representations. Cross-lagged analysis of both constructs during such a phase (for example by means of random intercept-, lag2 cross-lagged-, change-, our outcome-wide approaches and study designs; Klopack & Wickrama, 2020; Lüdtke & Robitzsch, 2022; VanderWeele et al., 2020) might yield insights into their longer-term developmental interplay. In a more controlled lab-based design, learners could be asked to work on experiments that make use of multiple representations. The shorter-term dynamic interplay between the two constructs might then be gauged for example by eye tracking. Since gaze data are assumed to allow interferences about a subject’s attention allocation and cognitive processes (van Gog & Jarodzka, 2013), prospective studies can use eye tracking to capture learners’ representational competence in action. This could be complemented by repeated assessments capturing development in learners’ conceptual knowledge. Moreover, analyzing gaze behavior could shed light on different task-solving processes occurring in males vs. females or high- vs. low-performing subjects. Prior research attributes gender differences in spatial ability to gender-specific strategies in processing visuo-spatial information (Kramer et al., 1996). For the purpose of methodological triangulation (Denzin, 2012), future studies could also collect verbal data to improve the interpretation of gaze data. Cued retrospective reports for which subjects are shown their own eye movements recordings to explain why they looked on specific task areas in a certain order seem to be a promising future approach as they do not affect performance or data quality (Holmqvist et al., 2011). The uncovering of gender-specific or experts’ task-solving processes, particularly regarding the representational knowledge test, could further be used to derive supportive instructions for low-performing students.

Since students in our sample self-selected into their studies, we do also not know about the generalizability of gender differences and our other findings to other populations. It would be informative to examine the generalizability is samples that are more heterogeneous regarding characteristics such as socioeconomic status and prior educational experiences. Although electromagnetism is a standard topic in high school Physics-curricula, some teachers still might have skipped this topic. Future studies should assess learners’ actual experience with this topic to be better able to interpret their content knowledge-data. In addition, analyses of our data could further compare our findings across our four samples of students (the data set is publicly available from the link indicated under Malone et al., 2021). This might also include analyses of measurement invariance to examine to which extent the employed measures show similar or different psychometric structure across students with different backgrounds. Gender differences were only briefly compared between samples in the present manuscript, since this was not in the focus of our research questions. This might be done in more detail in future research, to examine how differences in learners’ background relate to potential differences in gender differences across samples.

Although we corrected the standard errors of correlation estimates for clustering within samples and for non-normality, manifest correlations still contain measurement error and, from the perspective of causality, confounding. We still reported and interpreted manifest correlations since simple, unadjusted correlations connect well to our research question and the estimand that we were interested in examining (Lundberg et al., 2021). Also, other studies and meta-analyses commonly prefer unadjusted correlations over multiple regression when a simple relation is in the focus of research (e.g., Edelsbrunner et al., 2022; Schneider et al., 2017, 2018).

Finally, we had too few students who indicated a diverse category to include them in the gender-specific statistical models. It should be examined how learners who identify with this category compare to those within the other categories, and whether they need similar or different instructional support (Fisher et al., 2021). Descriptive statistics in our sample showed average representational competence yet low conceptual knowledge within this group of students, indicating that they might particularly require support in building up conceptual knowledge.

## Conclusion

Our results show that conceptual knowledge and representational competence are separable constructs on the one hand but are also clearly interrelated on the other. Even though their mutual interplay still needs to be investigated in more detail, it would not be going too far out on a limb to demand that science education be designed in such a way that it explicitly promotes both. If representational competence is considered an independent knowledge component that is at least in parts contextually independent, it could also be addressed in interdisciplinary teaching.

The second important result of our work is the difference found between females and males, both in the mean scores for conceptual knowledge and representational competence, but also in the strength of the relation of the two constructs, which was lower for females. Future studies should focus on the processes responsible for females experiencing difficulties in using provided representations for understanding the represented concepts. Findings should then be used as the basis for developing and evaluating instructional approaches to promote (meta-) representational competence for all students.

## Supplementary Information

**Additional file 1: Table S1.** Detailed descriptive statistics of central study measures. **Table S2.** Item statistics for representational competence-test. **Table S3.** Item statistics for conceptual knowledge-test. **Table S4.** Gender-specific correlations across samples.

## Data Availability

All data and materials are openly available from Malone et al. (2021).
