# Supplementary material for: The relation of representational competence and conceptual knowledge in female and male undergraduates
Source: Int J STEM Educ. 2023 Jun 21;10(1):44. doi: 10.1186/s40594-023-00435-6 (PMC10285021; doi:10.1186/s40594-023-00435-6)
Supplement: Supplementary file 1 — Additional file 1: Table S1. Detailed descriptive statistics of central study measures. Table S2. Item statistics for representational competence-test. Table S3. Item statistics for conceptual knowledge-test. Table S4. Gender-specific correlations across samples. [file 40594_2023_435_MOESM1_ESM.docx]

**The Relation of Representational Competence and Conceptual Knowledge in Female and Male Undergraduates**

**Additional file 1**

| Table S1: | Detailed Descriptive Statistics of Central Study Measures | 2 |
| --- | --- | --- |
| Table S2: | Item Statistics for Representational Competence-Test | 3 |
| Table S3 | Item Statistics for Conceptual Knowledge-Test | 4 |
| Table S4 | Gender-Specific Correlations Across Samples | 5 |

**Table S1**

*Detailed Descriptive Statistics of Central Study Measures*

|  | Representational competence (max. = 12) | Conceptual knowledge (max. = 12) |
| --- | --- | --- |
| M | 6.38 | 3.6 |
| SD | 2.9 | 3.19 |
| Min | 0 | 0 |
| Max | 12 | 12 |
| Range | 12 | 12 |
| Median | 6 | 3 |
| Mode | 4 | 1 |
| Skewness | 0.2 | 0.99 |
| Kurtosis | -0.9 | 0.08 |

**Table S2**

*Item Statistics for Representational Competence-Test*

| Item | *M* (% solved) | Item-total *r* | Item-rest *r* | Lambda | Lambda std. |
| --- | --- | --- | --- | --- | --- |
| 1 | 0.36 | 0.44 | 0.38 | 1.00 | 0.55 |
| 2 | 0.83 | 0.38 | 0.33 | 1.12 | 0.62 |
| 3 | 0.82 | 0.30 | 0.26 | 0.77 | 0.42 |
| 4 | 0.31 | 0.41 | 0.36 | 1.10 | 0.60 |
| 5 | 0.33 | 0.46 | 0.41 | 1.07 | 0.58 |
| 6 | 0.76 | 0.28 | 0.23 | 0.57 | 0.31 |
| 7 | 0.31 | 0.58 | 0.49 | 1.14 | 0.63 |
| 8 | 0.44 | 0.59 | 0.50 | 1.15 | 0.63 |
| 9 | 0.56 | 0.59 | 0.50 | 1.41 | 0.77 |
| 10 | 0.62 | 0.62 | 0.53 | 1.46 | 0.80 |
| 11 | 0.53 | 0.28 | 0.24 | 0.72 | 0.39 |
| 12 | 0.51 | 0.49 | 0.43 | 1.19 | 0.66 |
| *Note*. Item-total r = estimated correlation of item with total scale score; Item-rest r = estimated correlation of item with score of remaining items. Lambda = unstandardized factor loading-estimate from confirmatory factor analysis; Lambda std. = standardized factor loading. | | | | | |

**Table S3**

*Item Statistics for Conceptual Knowledge-Test*

| Item | *M* (% solved) | Item-total *r* | Item-rest *r* | Lambda | Lambda std. |
| --- | --- | --- | --- | --- | --- |
| 1 | 0.35 | 0.62 | 0.57 | 1.00 | 0.75 |
| 2 | 0.47 | 0.55 | 0.50 | 0.90 | 0.68 |
| 3 | 0.30 | 0.65 | 0.59 | 1.13 | 0.85 |
| 4 | 0.31 | 0.58 | 0.52 | 1.03 | 0.78 |
| 5 | 0.29 | 0.60 | 0.55 | 1.02 | 0.76 |
| 6 | 0.40 | 0.46 | 0.42 | 0.76 | 0.57 |
| 7 | 0.16 | 0.33 | 0.30 | 0.69 | 0.52 |
| 8 | 0.43 | 0.51 | 0.46 | 0.85 | 0.64 |
| 9 | 0.19 | 0.53 | 0.49 | 0.95 | 0.71 |
| 10 | 0.21 | 0.55 | 0.49 | 0.93 | 0.70 |
| 11 | 0.20 | 0.50 | 0.45 | 0.82 | 0.62 |
| 12 | 0.29 | 0.59 | 0.53 | 0.97 | 0.73 |
| *Note*. Item-total r = estimated correlation of item with total scale score; Item-rest r = estimated correlation of item with score of remaining items. Lambda = unstandardized factor loading-estimate from confirmatory factor analysis; Lambda std. = standardized factor loading. | | | | | |

**Table S4**

*Correlation of Representational Competence and Conceptual Knowledge Separate for Females and Males in the Different Samples*

| Sample | Females | Males |
| --- | --- | --- |
| 1 | .26 [.03; .46] | .64 [.49; .75] |
| 2 | .03 [-.30; .35] | .45 [.22; .63] |
| 3 | .36 [-.02; .65] | .42 [.09; .67] |
| 4 | .47 [.36; .57] | .55 [.45; .64] |

*Note.* Descriptions of samples by numbers provided in method-section.
